# In Situ Construction of ZnO/Ni_2_S_3_ Composite on Ni Foam by Combing Potentiostatic Deposition with Cyclic Voltammetric Electrodeposition

**DOI:** 10.3390/mi12070829

**Published:** 2021-07-16

**Authors:** Sa Lv, Peiyu Geng, Huan Wang, Fan Yang, Jia Yang, Chao Wang, Yaodan Chi, Xiaotian Yang

**Affiliations:** Key Laboratory for Comprehensive Energy Saving of Cold Regions Architecture of Ministry of Education, Jilin Provincial Key Laboratory of Architectural Electricity & Comprehensive Energy Saving, Jilin Jianzhu University, Changchun 130118, China; gengpeiyu1@163.com (P.G.); whuan@ciac.ac.cn (H.W.); ctpnxn@163.com (F.Y.); yangjia@jlju.edu.cn (J.Y.); wangchao@jlju.edu.cn (C.W.); chiyaodan@jlju.edu.cn (Y.C.)

**Keywords:** electrode material, ZnO, Ni foam, cyclic voltammetric electrodeposition

## Abstract

The ZnO/Ni_2_S_3_ composite has been designed and in situ synthesized on Ni foam substrate by two steps of electrodeposition. ZnO was achieved on Ni foam by a traditional potentiostatic deposition, followed by cyclic voltammetric (CV) electrodeposition, to generate Ni_2_S_3_, where the introduction of ZnO provides abundant active sites for the subsequent Ni_2_S_3_ electrodeposition. The amount of deposit during CV electrodeposition can be adjusted by setting the number of sweep segment and scan rate, and the electrochemical characteristics of the products can be readily optimized. The synergistic effect between the ZnO as backbones and the deposited Ni_2_S_3_ as the shell enhances the electrochemical properties of the sample significantly, including a highly specific capacitance of 2.19 F cm^−2^ at 2 mA cm^−2^, good coulombic efficiency of 98%, and long-term cyclic stability at 82.35% (4000 cycles).

## 1. Introduction

As an important energy storage device, the supercapacitor has attracted more and more attention by virtue of the advantages of its fast charging speed, long cycle life, large current discharge capacity, high power density, and friendly environment [1]. As the most critical part of the supercapacitor, electrode materials are the primary factor to determine the performance of the device. Researchers concentrate their efforts on the design and regulation of the composition, structure and morphology of the electrode materials, so as to realize the optimization of the properties of the device [2,3].

There are two main ways to regulate the structure and morphology of electrode materials. The first is to exploit different preparation methods, such as hydrothermal reaction, electrodeposition technique, and in situ polymerization processes. It also includes the selection of different electrode substrates, such as Ni foam, Cu foam, and carbon cloth, to directly control the structure and morphology of the electrode materials [4]. Another way is to select an appropriate component to construct the composite structure [5], which can be divided into two cases. In one case, the components with capacitance characteristics constitute the composite materials, and this can generate a synergistic effect between the two active materials and the performance of materials can be improved thereby. In the other case, the component without capacitance characteristics acts as an effective additive ingredient to increase the conductivity, or as a backbone to provide sufficient surface area for the recombination of other components. For example, He et al. fabricated CuO@Ni–Fe-layered double hydroxide (LDH) nanorods arrays on Cu foam, by two-step in situ electrochemical processes. The CuO nanorods provide support for the subsequent electrodeposition of Ni–Fe LDH, and also participate in the electrochemical reaction as an active component, which reveals an improved specific capacitance of 2.682 F cm^−2^, responding to the scan rates of 2 mV s^−1^ [6]. Yuksel et al. designed a Ag nanowire@Ni(OH)_2_ coaxial nanocomposite electrode. The introduction of Ag improves the electrical conductivity of the material, and synergistically promotes ion and electron transport with Ni(OH)_2_, which reveals an enhanced specific capacitance of 1165.2 F g^−1^ at a current density of 3 A g^−1^ [7]. Both copper oxide and Ag have their own characteristics. For instance, copper has various valence states of oxides, such as Cu(OH)_2_, CuO, and Cu_2_O. Therefore, when copper oxides are used as electrode material, we should pay attention to analyze the valence state of copper, because their energy storage mechanisms are different. Although Ag can be used as an active component, to participate in the electrochemical reaction, the encapsulation by other components causes the decline in the electrical properties of Ag.

Based on the above analysis, ZnO has also been explored as a component of electrode materials, because of its convenient preparation and controllable morphology. Generally, researchers have prepared ZnO-based composite electrode materials by a two-step method, such as ZnO@Ni(OH)_2_ [8], ZnO@MnO_2_ [9], ZnO@Co(OH)_2_ [10], and ZnO@CoFe_2_O_4_ [11]. All of these achieved stable ZnO nanowires [8], nanorods [9], nanopets [10], or nanoplates [11], play the role of an active component of the composite electrode material, and provide large specific surface area for the following growth of other components. The typical preparation methods include the hydrothermal method, seed-layer growth, and chemical bath deposition [12,13]. On the other hand, Ni(OH)_2_ is one of the most ideal electrode materials, with abundant storage and high theoretical specific capacitance [14]. Therefore, among ZnO- and Ni-based compounds, ZnO/Ni(OH)_2_ is relatively more explored [15]. In order to expand the selection range of electrode materials, Ni-base sulfide has gradually attracted more and more attention. As a new electrode material, metal sulfide not only has a complex structure, outstanding physical properties, and large specific capacitance, but also has low electronegativity, which is beneficial for electron transport [16,17].

In this paper, the ZnO/Ni_2_S_3_ composite was in situ grown on Ni foam substrate, by a two-step electrodeposition, including a traditional potentiostatic deposition and a new deposition strategy, cyclic voltammetric (CV) electrodeposition. Compared with the pure Ni_2_S_3_, the introduction of ZnO improves the electrical conductivity of the material and provides abundant active sites for the subsequent Ni_2_S_3_ electrodeposition. As a binder-free electrode material for supercapacitor, the ZnO/Ni_2_S_3_ composite exhibits enhanced storage properties.

## 2. Materials and Methods

### 2.1. Materials

Ni foam (100 PPI, Kunshan Dessco CO., Ltd, Suzhou, Jiangsu, China) was cleaned with acetone, ethanol and deionized water repeatedly. The reagents used in the experiment were purchased from Sinopharm Chemical Reagent Co., Ltd. (Shanghai, China) including zinc nitrate hexahydrate (Zn(NO_3_)_2_·6H_2_O), potassium chloride (KCl), nickel chloride hexahydrate (NiCl_2_·6H_2_O), thiourea (CH_4_N_2_S) and sodium hydroxide (NaOH).

### 2.2. Preparation of ZnO/Ni_2_S_3_ Composite Electrode

In the first step, the mixed solution of 0.2 M Zn(NO_3_)_2_ and 4.8 M KCl was prepared and used as electrolyte to electrodeposit ZnO by exploiting a three-electrode system. Ni foam was used as working electrode, saturated calomel electrode (SCE) was used as reference electrode, and a Pt plate was used as counter electrode, respectively. Potentiostatic electrodeposition technique was adopted to perform 90 s at −10 V versus SCE.

**Figure 1:**
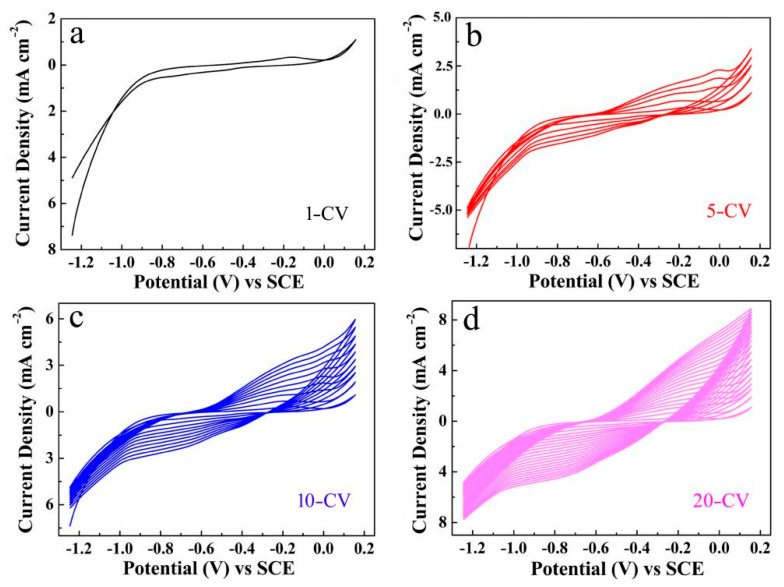
The CV curves of Ni_2_S_3_ electrodeposition on the ZnO/Ni with different cycles: (**a**) 1-CV; (**b**) 5-CV; (**c**) 10-CV; (**d**) 20-CV.

The second step for Ni_2_S_3_ electrodeposition was carried out with the same three-electrode system, except the working electrode is Ni foam deposited by ZnO obtained in the previous step and the electrolyte is a mixture solution containing 0.05 M NiCl_2_ and 1 M CH_4_N_2_S. The CV electrodeposition was performed by 10 cycles (abbreviated as 10-CV) in the range of −1.25 to 0.16 V at a scan rate of 0.005 V s^−1^. The CV electrodeposition curves obtained from different cycles are labeled as 1-CV, 5-CV, 10-CV and 20-CV (Figure 1). The corresponding products are to be tested after rinsing thoroughly.

### 2.3. Characterization

X-ray diffraction (XRD Cu Kα radiation with λ = 1.5406 Å), field-emission scanning electron microscopy FE-SEM (JSM-7610F, Tokyo, Japan), and X-ray photoelectron spectroscopy (XPS ESCALAB 250Xi, ThermoFisher Scientifific Co., Ltd, Waltham, MA, USA Al Kα as X-ray source) were used to characterize the product. A CHI 760E electrochemical workstation was used to conduct the electrochemical tests, including cyclic voltammetry (CV), galvanostatic charge–discharge (GCD) and cycling performance, at a three-electrode system in 2 M NaOH aqueous solution, in which the ZnO/Ni_3_S_2_ on Ni foam was served as working electrode, and a Pt plate and Hg/HgO electrode were served as the counter electrode and reference electrode, respectively.

## 3. Results

**Figure 2:**
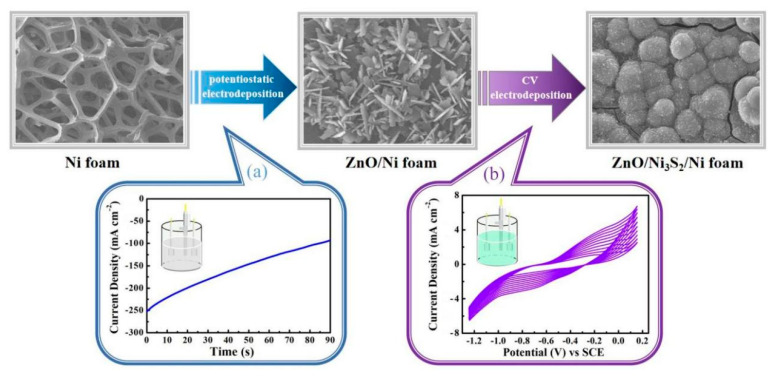
Schematic illustration of the in situ construction process of ZnO/Ni_2_S_3_ composite: (**a**) potentiostatic electrodeposition; (**b**) CV electrodeposition.

The ZnO/Ni_2_S_3_ composite is synthesized on Ni foam substrate by a two-step electrodeposition, and the construction process is illustrated in Figure 2. Firstly, potentiostatic electrodeposition is adopted to deposit ZnO nanosheets on the surface of Ni foam substrate. After that, CV electrodeposition is employed, by using a similar three-electrode system to achieve a Ni_2_S_3_ layer on the surface of ZnO, and the thickness of the Ni_2_S_3_ layer increases gradually with the increase in the number of cycles.

**Figure 3:**
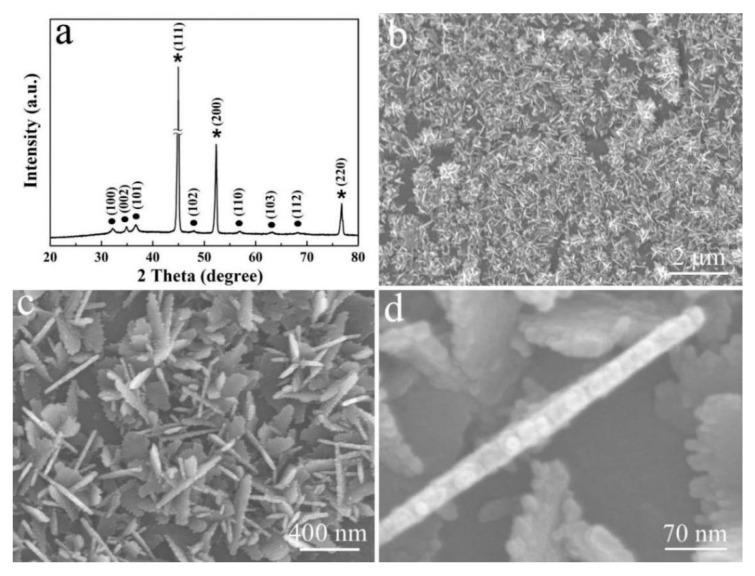
(**a**) XRD pattern and (**b**–**d**) FE-SEM images of ZnO nanosheets formed on Ni foam substrate at different magnifications.

The XRD pattern of the product that is electrodeposited on the Ni foam substrate by the potentiostatic deposition method is shown in Figure 3a. Ni foam shows three strong diffraction peaks, which are marked with an asterisk (JCPDS card No. 03-1051). All other diffractions, marked with dot, are in good agreement of (100), (002), (101), (102), (110), (103), and (112) planes of the hexagonal phase of ZnO (JCPDS No.03-0888). The morphology and structure of ZnO are presented in Figure 3b–d. The FE-SEM image, in Figure 3b, displays that the dense ZnO nanostructures are uniformly distributed on the Ni foam substrate. The corresponding enlarged image in Figure 3c confirms that the ZnO nanosheets cross with each other, and the edges of these nanosheets are serrated. Closer observation reveals that the nanosheets are ca. 22 nm thick, and the serrated edges confirm that these nanosheets are composed of ZnO particles, with a size of about 15 nm (Figure 3d).

**Figure 4:**
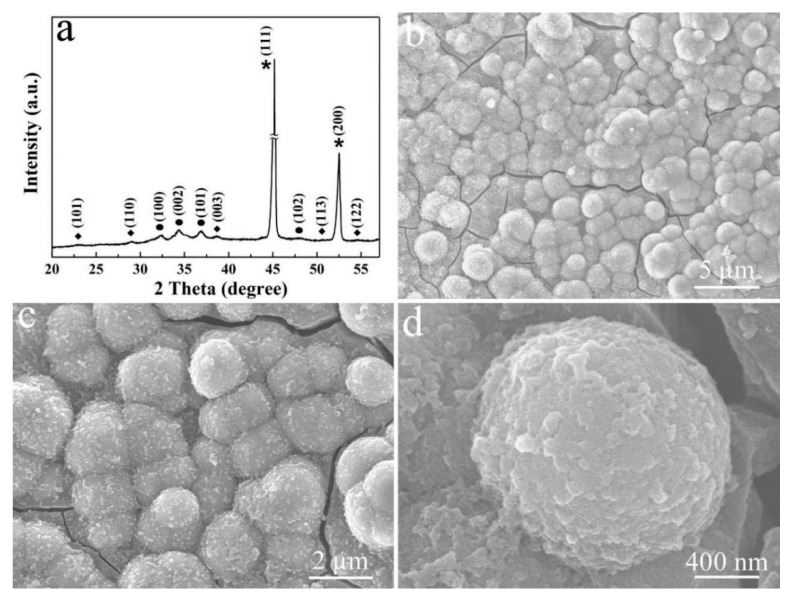
(**a**) XRD pattern and (**b**–**d**) FE-SEM images of ZnO/Ni_2_S_3_ composite at different magnifications.

The ZnO/Ni_2_S_3_ composite was further obtained through the subsequent CV electrodeposition, as shown in Figure 4a. The XRD pattern of the product exhibits three kinds of diffraction peaks. In addition to the peaks that are marked with asterisks and dots belong to the Ni foam substrate and ZnO, respectively, the peaks that are marked with diamonds belong to the Ni_2_S_3_ obtained by CV electrodeposition (JCPDS card No. 44-1418). Figure 4b–d are the corresponding FE-SEM images, as seen from Figure 4b, ZnO nanosheets are completely covered and wrapped into ZnO/Ni_2_S_3_ composite nanospheres. The enlarged image in Figure 4c confirms that these ZnO/Ni_2_S_3_ composite nanospheres have rough surfaces, with approximately 1.4–1.8 µm in diameter. In fact, the rough surface is made up of tiny Ni_2_S_3_ particles that have been electrodeposited layer by layer (Figure 4d).

**Figure 5:**
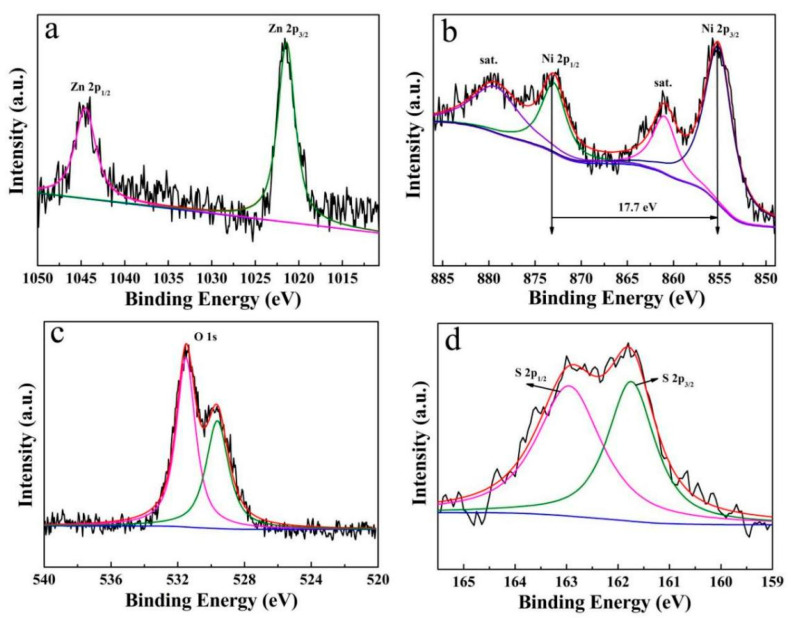
XPS spectra of ZnO/Ni_2_S_3_ composite: (**a**) Zn 2p; (**b**) Ni 2p; (**c**) O 1s; (**d**) S 2p.

Figure 5 depicts the survey XPS spectra of the ZnO/Ni_2_S_3_ composite. From the Zn 2p spectrum (Figure 5a), two peaks located at 1044.70 and 1021.40 eV belong to Zn 2p_1/2_ and Zn 2p_3/2_, respectively, confirming the existence of ZnO [10,18]. As for the Ni 2p spectrum in Figure 5b, two major peaks of the binding energies at 855.60 and 873.30 eV, with a spin-energy separation of 17.7 eV, correspond to Ni 2p_3/2_ and Ni 2p_1/2_, respectively, indicating the emergence of Ni^2+^ and Ni^3+^ [19,20,21]. Apart from that, both of them have a satellite peak at 861.20 and 879.80 eV [22]. In regard to the O 1s spectrum in Figure 5c, as previously reported, the binding energy of 531.20 eV is ascribed to the oxygen bond with Zn^2+^, and the binding energy of 529.90 eV is assigned to the oxygen of NiO, which was generated from the reduction in part of the Ni^3+^ during electrodeposition [10,18]. The S 2p spectrum in Figure 5d can be divided into two peaks that are centered at 162.99 and 161.54 eV, corresponding to S 2p_1/2_ and S 2p_3/2_ of S^2−^, respectively, which are in agreement with those of Ni_2_S_3_ in the reports [23,24].

**Figure 6:**
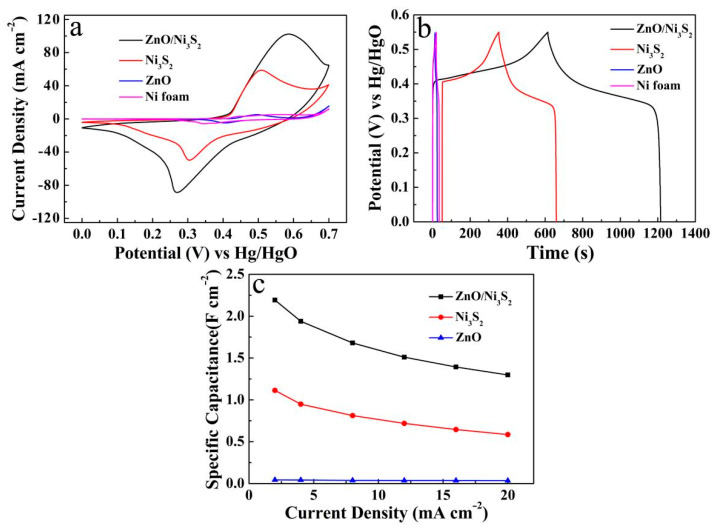
(**a**) CV curves at 20 mV s^−1^; (**b**) GCD curves and (**c**) line diagrams of specific capacitance at different current densities of ZnO/Ni_2_S_3_, Ni_2_S_3_, ZnO electrode and Ni foam substrate.

In order to study the electrochemical properties of the ZnO/Ni_2_S_3_ composite, CV and GCD measurements were carried out in a three-electrode system, in 2 M NaOH aqueous solution. As seen in Figure 6a, the CV curves for various electrodes are compared, at a scan rate of 20 mV s^−1^, including ZnO/Ni_2_S_3_, Ni_2_S_3_, ZnO, and Ni foam substrate. The ZnO/Ni_2_S_3_ composite has the largest enveloping area compared with the others, proving the maximum specific capacitance, while the ZnO and Ni foam have almost no capacitance characteristics. Further observation reveals that the CV curve of the ZnO/Ni_2_S_3_ composite contains a pair of strong redox peaks in the potential range of 0–0.7 V, which is ascribed to Ni^2+^/Ni^3+^ as the following electrochemical reaction [21,25]:

(1) $$Ni_{3}S_{2}+3{\mathrm{OH}}^{-}↔Ni_{3}S_{2}{\left(\mathrm{OH}\right)}_{3}+3e^{-}$$

Figure 6b depicts the GCD curves corresponding to the four electrodes at 2 mA cm^−2^. Among them, the ZnO/Ni_2_S_3_ composite has the longest discharge time, and thus the largest specific capacitance. The test results are consistent with those of the CV measurement. Figure 6c compares the line diagrams of the specific capacitance of the three electrodes at different discharge current densities, revealing that the significant enhancement of the electrochemical properties of the ZnO/Ni_2_S_3_ composite is attributed to the introduction of ZnO, although ZnO has almost no capacitance characteristics.

ZnO is grown on Ni foam by potentiostatic electrodeposition, based on the following reaction [9]:

(2) $$2{\mathrm{Zn}}^{2+}+O_{2}+4e^{-}\to 2\mathrm{ZnO}$$

**Figure 7:**
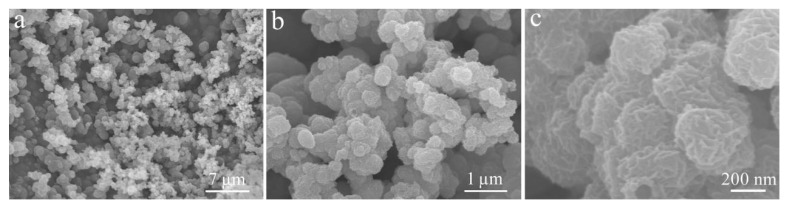
FE-SEM images of Ni_2_S_3_ electrodeposited on the Ni foam substrate in the absence of Zn. FE-SEM images at (**a**) 7 µm, (**b**) 1 µm, (**c**) 200 nm.

In the absence of ZnO, Ni_2_S_3_ nanoparticles are piled in disorder on the Ni foam substrate, and the surface is wrinkled (Figure 7a–c). In comparison, the pre-formed ZnO as backbones provides support and abundant active sites for the subsequent electrodeposition of Ni_2_S_3_.

In view of the role of Ni_2_S_3_, it is necessary to investigate the effects of different CV electrodeposition cycles of Ni_2_S_3_ on the morphology and electrochemical properties of the ZnO/Ni_2_S_3_ composite. The reaction of sulfidization of nickel salt can be described as follows [20]:

(3) $${\left({\mathrm{NH}}_{2}\right)}_{2}\mathrm{CS}+2H_{2}O=H_{2}S+{\mathrm{CO}}_{2}+2{\mathrm{NH}}_{3}$$

(4) $$2H_{2}S+3{\mathrm{NiCl}}_{2}={\mathrm{Ni}}_{3}S_{2}+4\mathrm{HCl}$$

**Figure 8:**
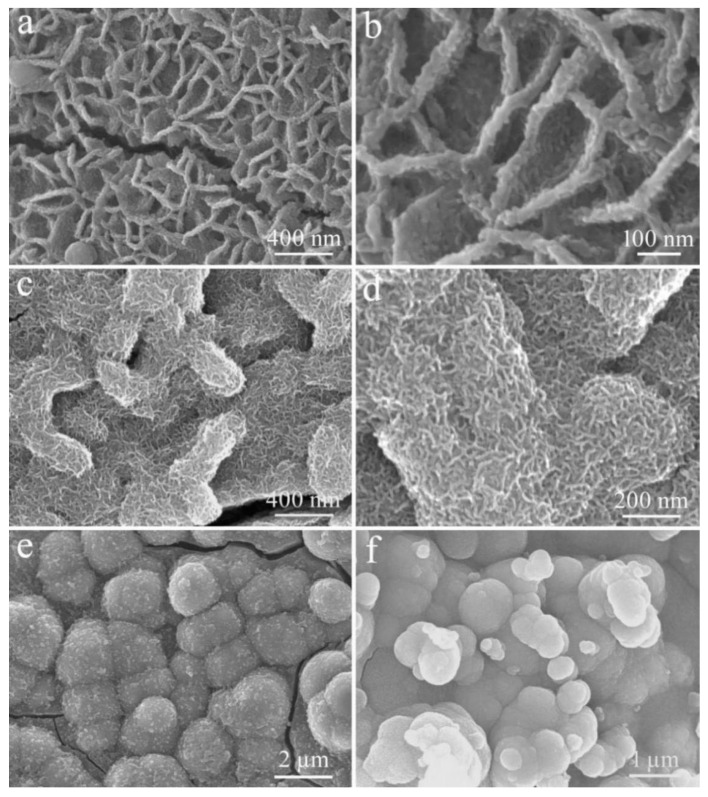
FE-SEM images of the ZnO/Ni_2_S_3_ composite grown on Ni foam substrate with different CV electrodeposition cycles of Ni_2_S_3_: (**a**,**b**) 1-CV; (**c**,**d**) 5-CV; (**e**) 10-CV; (**f**) 20-CV.

When the CV electrodeposition was performed for one circle (1-CV), the ZnO nanosheets are covered with a thin layer of Ni_2_S_3_, but the original shape of the ZnO nanosheet is clearly visible (Figure 8a). Figure 8b presents the surface and edges of these ZnO nanosheets, which are roughened because of the coating of the Ni_2_S_3_ nanoparticles. When the electrodeposition reaches 5-CV, as seen in Figure 8c,d, the morphology of these ZnO nanosheets is basically completely covered, and only the edges of these nanosheets, which become wider, due to the coverage of Ni_2_S_3_, can be vaguely observed. When the electrodeposition is extended to 10-CV, the nanospheres with a diameter of 1.4–1.8 µm are formed, because of the continuous deposition of Ni_2_S_3_, as shown in Figure 8e. The electrodeposition of up to 20-CV results in the disordered accumulation of Ni_2_S_3_ particles.

**Figure 9:**
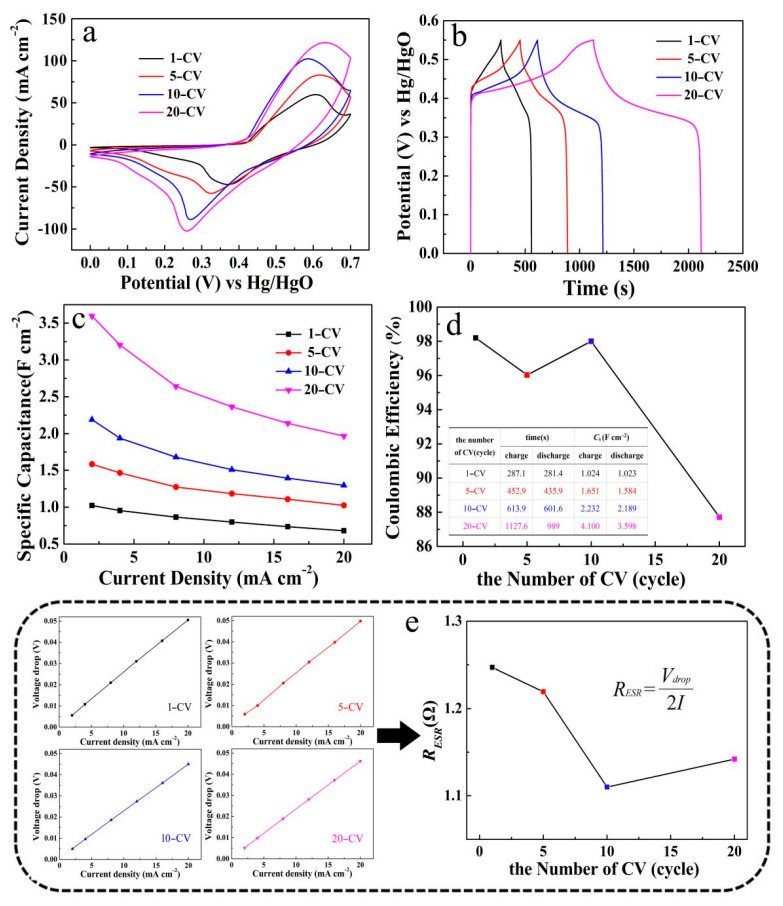
(**a**) CV curves, (**b**) GCD curves, (**c**) line diagrams of specific capacitance at different current densities, (**d**) coulombic efficiency and (**e**) voltage drops and *R*_ESR_ of the ZnO/Ni_2_S_3_ composite grown on Ni foam substrate with different CV electrodeposition cycles.

Figure 9 records the electrochemical performance comparison charts of the ZnO/Ni_2_S_3_ composite with different CV electrodeposition cycles of Ni_2_S_3_, including 1-CV, 5-CV, 10-CV, and 20-CV. Figure 9a–c display the CV curves, GCD curves, and the line diagrams of specific capacitance at different current densities. The test results confirm that the electrochemical performances of 1-CV to 20-CV are gradually enhanced with the increasing of the number of Ni_2_S_3_ electrodeposition cycles. However, from the perspective of coulombic efficiency (Figure 9d), the efficiency of 1-CV and 10-CV is about 98% at a discharge current density of 2 mA cm^−2^; They are relatively high compared with the others. Furthermore, the line diagram of the average *R*_ESR_ in Figure 9e, which is deduced from the voltage drops during the GCD tests, demonstrates that the *R*_ESR_ of 10-CV is the lowest. From the above test data results, two trends can be inferred, with the increase in the number of electrodeposition cycles of Ni_2_S_3_. On one hand, the amount of Ni_2_S_3_ that is electrodeposited inevitably increases, which reflects the enhanced CV and GCD characteristics. On the other hand, the skeleton supporting effect of ZnO is weakened, or even disappeared. Meanwhile, the excessive deposition leads to the disordered accumulation of particles. Therefore, we choose 10-CV as the electrodeposition cycles of Ni_2_S_3_ for further in-depth study.

**Figure 10:**
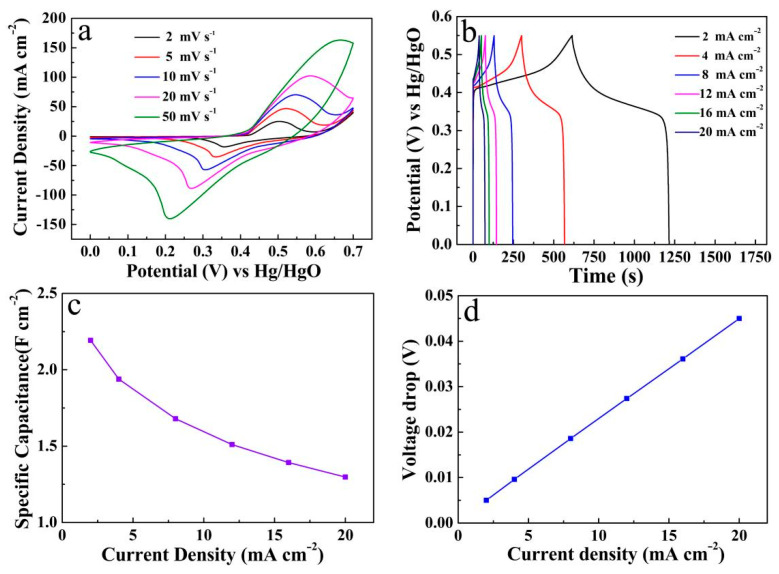
(**a**) CV curves, (**b**) GCD curves, (**c**) line diagram of specific capacitance at different current densities and (**d**) voltage drops of the ZnO/Ni_2_S_3_ composite (10-CV) grown on Ni foam substrate.

For the ZnO/Ni_2_S_3_ composite (10-CV), further CV measurements were performed, at different scan rates of 2–50 mV s^−1^ in the same potential window, as shown in Figure 10a. The redox peaks are symmetrical, and the variation trend of the CV curves at different scan rates is basically the same. In addition, with the increase in the scan rate, the area surrounded by the CV curves gradually increases, while the specific capacitance gradually decreases. This decrease is attributed to the fact that some active sites cannot fully participate in the redox reaction, due to the restriction of ion/electron diffusion at a larger scan rate [7]. Figure 10b reveals the GCD measurements at various current densities of 2–20 mA cm^−2^, in a potential range of 0–0.55 V. The GCD curves show an obvious pseudocapacitance characteristic because of their nonlinearity. The coulombic efficiency is as high as 98% at 2 mA cm^−2^, and detailed data have been recorded in Figure 9d. In addition, the specific capacitance of the ZnO/Ni_2_S_3_ electrode can be calculated by the formula in the Supporting Information [26].

As seen from Figure 10c, the line diagram of specific capacitance values of the ZnO/Ni_2_S_3_ electrode are about 2.19, 1.94, 1.68, 1.51, 1.39, and 1.30 F cm^−2^, corresponding to discharge current densities of 2, 4, 8, 12, 16, 20 mA cm^−2^, respectively. Meanwhile, according to Equation (6), the average *R*_ESR_ is 1.11 Ω cm^−2^, as shown in Figure 10d. In addition, the CV curves, and GCD curves, and the corresponding line diagram of the specific capacitance at different current densities of Ni_2_S_3_ and ZnO, were also carried out for comparison in Figure S1.

**Figure 11:**
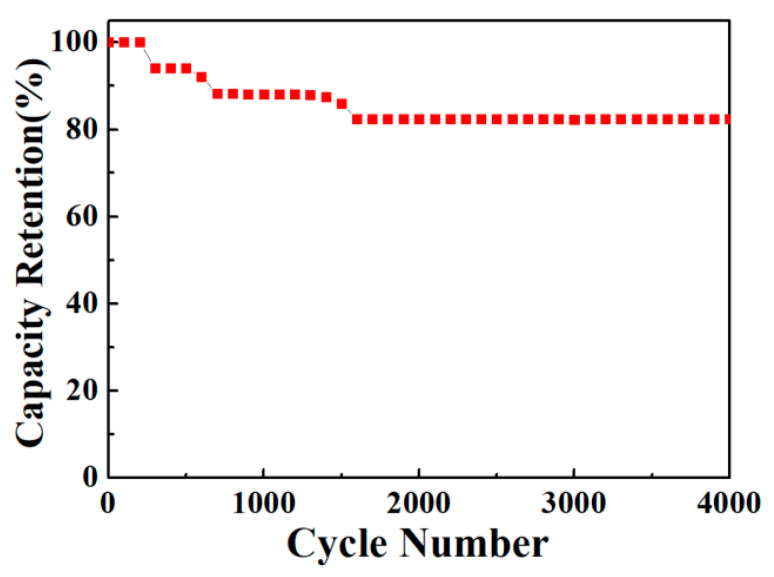
Cycling performance of the ZnO/Ni_2_S_3_ electrode.

The cyclic stability of the ZnO/Ni_2_S_3_ composite electrode is revealed in Figure 11. The specific capacitance is kept at the original value of 88.10% after 1000 cycles, and stabilizes the value at 82.35% within the range of 4000 cycles. After the cycle test, the general structure of the ZnO/Ni_2_S_3_ composite remains almost unchanged (Figure S2).

The in situ two-step electrodeposition of the ZnO/Ni_2_S_3_ composite presents two aspect distinct advantages. On one hand, from the perspective of the preparation method, compared with the hydrothermal method and seed-layer growth method, electrodeposition deposition technology has the advantages of a convenient operation process, shorter experiment period, and good repeatability. In particular, this CV electrodeposition method, compared with the traditional constant-voltage deposition and constant-current deposition, has more adjustable parameters and a more intuitive deposition curve [27]. At the same time, the in situ electrodeposition strategy decreases the contact resistance and provides an unobstructed path for the transmission of ions/electrons [10]. On the other hand, from the point of view of composition selection for electrode materials, ZnO has the characteristics of a controllable and diverse structure and morphology, and friendly environment [15]. The introduction of ZnO provides support for the subsequent Ni_2_S_3_ electrodeposition. In addition, Ni_2_S_3_ has more research space compared with the traditional Ni(OH)_2_, NiO- and Ni-based double hydroxide [19,21,23]. Therefore, the facile method can be promoted to realize the efficient regulation of other metal oxide-based composite and binary metal sulfides electrode materials.

## 4. Conclusions

The ZnO/Ni_2_S_3_ composite has been constructed and grown on Ni foam by combing potentiostatic electrodeposition and CV electrodeposition. Benefiting from the two-step in situ electrodeposition strategy, the ZnO/Ni_2_S_3_ electrode illustrated a highly specific capacitance value of 2.19 F cm^−2^ at 2 mA cm^−2^, and long-term cyclic stability at 82.35% (4000 cycles). This convenient method can be expanded for the preparation of other ZnO-based composite electrode materials, and binary or ternary metal sulfide electrode materials.

## Supplementary Materials

The following are available online at https://www.mdpi.com/article/10.3390/mi12070829/s1, Figure S1: CV curves, GCD curves and line diagram of specific capacitance at different current densities of Ni_3_S_2_ (a,c,e) and ZnO (b,d,f). Figure S2: FE-SEM images of the ZnO/Ni_3_S_2_ composite at different magnifications after electrochemical test. Equations for calculating specific capacitance.
