# Supplementary material for: In Situ Construction of ZnO/Ni2S3 Composite on Ni Foam by Combing Potentiostatic Deposition with Cyclic Voltammetric Electrodeposition
Source: Micromachines (Basel). 2021 Jul 16;12(7):829. doi: 10.3390/mi12070829 (PMC8306812; doi:10.3390/mi12070829)
Supplement: Supplementary file 1 [file micromachines-12-00829-s001.zip › micromachines-1272137-supplementary.pdf]

micromachines

Supplementary Materials

In Situ Construction of ZnO/Ni_2S_3 Composite on Ni Foam by Combing Potentiostatic Deposition with Cyclic Voltammetric Electrodeposition

Sa Lv *, Peiyu Geng, Huan Wang, Fan Yang, Jia Yang, Chao Wang, Yaodan Chi and Xiaotian Yang *

Key Laboratory for Comprehensive Energy Saving of Cold Regions Architecture of Ministry of Education, Jilin Provincial Key Laboratory of Architectural Electricity & Comprehensive Energy Saving, Jilin Jianzhu University, Changchun 130118, China; gengpeiyu1@163.com (P.G.); whuan@ciac.ac.cn (H.W.); ctpnxn@163.com (F.Y.); yangjia@jlju.edu.cn (J.Y.); wangchao@jlju.edu.cn (C.W.); chiyaodan@jlju.edu.cn (Y.C.)
* Correspondence: lvsa82@163.com (S.L.); hanyxt@163.com (X.Y.); Tel.: +86-0431-8456-6181 (S.L.)

Citation: Lv, S.; Geng, P.; Wang, H.; Yang, F.; Yang, J.; Wang, C.; Chi, Y.; Yang, X. In Situ Construction of ZnO/Ni_2S_3 Composite on Ni Foam by Combing Potentiostatic Deposition with Cyclic Voltammetric Electrodeposition. *Micromachines* **2021**, *12*, 829. https://doi.org/10.3390/mi12070829

Academic Editor: Hassan Karimi-Maleh

Received: 8 June 2021
Accepted: 12 July 2021
Published: 16 July 2021

Publisher's Note: MDPI stays neutral with regard to jurisdictional claims in published maps and institutional affiliations.

Copyright: © 2021 by the authors. Licensee MDPI, Basel, Switzerland. This article is an open access article distributed under the terms and conditions of the Creative Commons Attribution (CC BY) license (http://creativecommons.org/licenses/by/4.0/).

The areal capacitance of ZnO/Ni_3S_2 electrode is calculated according to the equation:

$$C_S = \frac{I \times \Delta t}{S \Delta V} \quad (1)$$

where Cs (F cm^{-2}) is the specific capacitance, I (A) is the charge–discharge current, Δt (s) is the discharging time, S (cm^2) is the effective area of the electrode and ΔV (V) represents the potential drop during discharge.

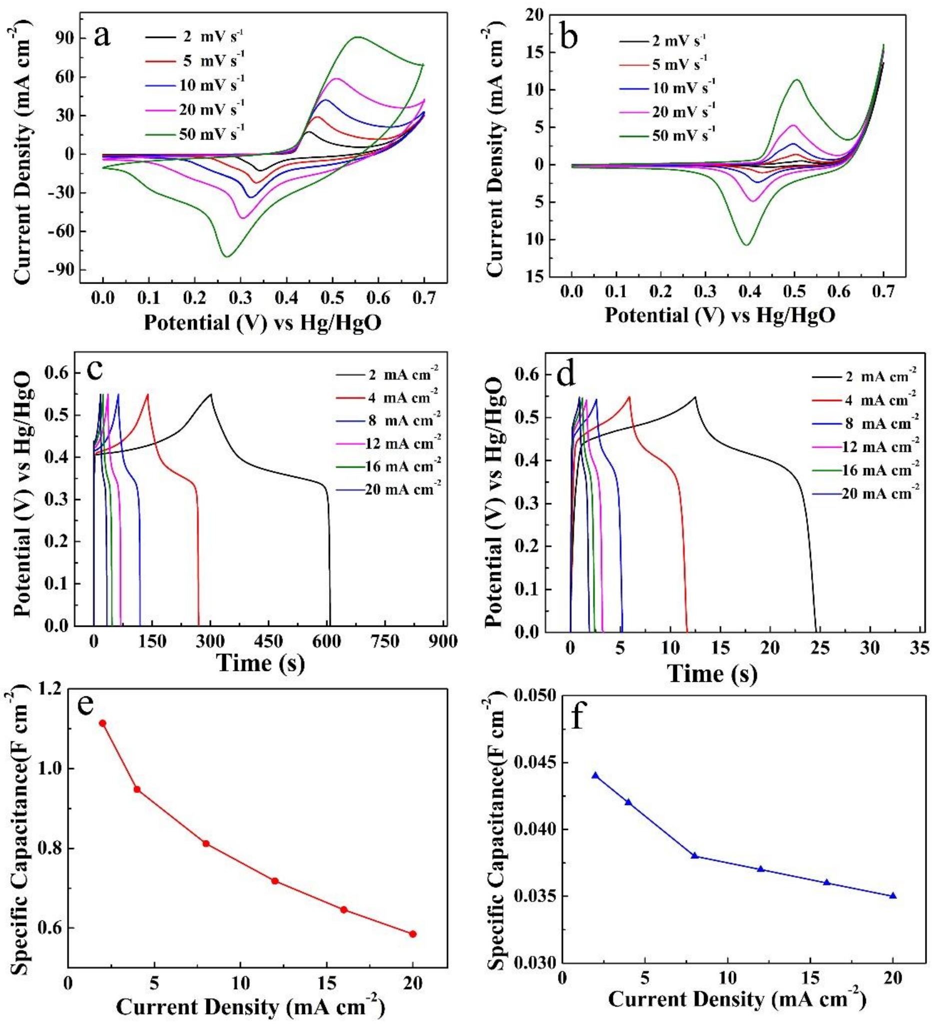

Figure S1. CV curves, GCD curves and line diagram of specific capacitance at different current densities of Ni_3S_2 (**a,c,e**) and ZnO (**b,d,f**).

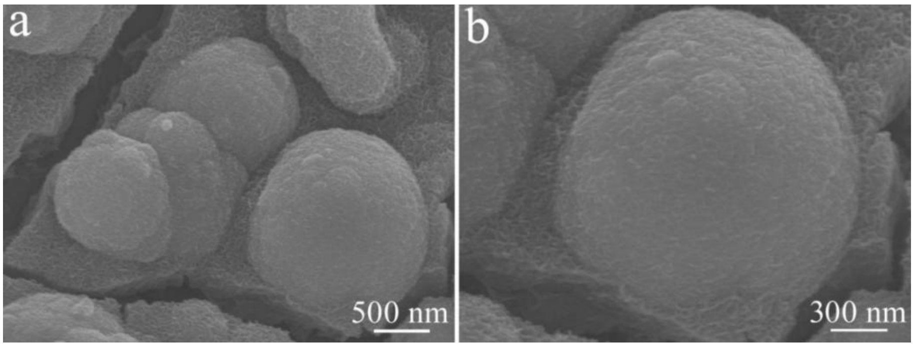

Figure S2. FE-SEM images of the ZnO/Ni_3S_2 composite at different magnifications after electrochemical test.
